# Nursing's Scientific Method

**DOI:** 10.1097/AJN.0000000000000167

**Published:** 2025-09-25

**Authors:** Mary Curry Narayan

**Affiliations:** **Mary Curry Narayan** is a founding member and an advisor at the International Home Care Nurses Organization (www.ihcno.org). Contact author: marycnarayan@gmail.com. The author has disclosed no potential conflicts of interest, financial or otherwise.

**Keywords:** critical thinking, evidence-based practice, nurse–patient collaboration, nursing process, nursing's scientific method, patient-centered care

## Abstract

The nursing process, developed by nursing theorist Ida Jean Orlando, has guided nurses for more than 65 years. Yet as nursing's responsibilities and evidence-based knowledge evolves, so should the nursing process. This article offers four recommendations to enhance the process: (1) changing “nursing process” to “nursing's scientific method”; (2) adding “relationship building” and “communication” to the method; (3) renaming “nursing diagnosis” and “outcome identification” to “problems/strengths identification” and “goals”; and (4) emphasizing that nursing's scientific method is a critical thinking methodology. These changes would better promote critical thinking, patient-centeredness, effectiveness of nursing care, and patient safety and engagement. Evidence and research are cited to support these recommendations.

During the late 1950s, nursing theorist Ida Jean Orlando began using evidence to develop the Nursing Process Theory.^1^ This was at a time when nurses were initiating efforts to transform nursing from a semiprofessional discipline into a truly independent, science-based profession. Originally, the nursing process consisted of four elements: assessment, planning, implementation, and evaluation. The American Nurses Association (ANA) endorsed the nursing process in its first *Standards of Nursing Practice*,^2^ identifying each of the four elements as a “standard of practice.” It later expanded the four elements to include “nursing diagnosis” and “expected outcomes.”^3^

Since its inception, the nursing process has become a standard in nursing education around the world,^4^-^8^ with the World Health Organization, the International Council of Nurses, and the Joint Commission supporting its use to promote good nursing care.^9^ Using the nursing process to guide thinking about how to best support patients leads to greater effectiveness of care and better outcomes.^10^ When providing patient care, nurses who do not use critical thinking and the nursing process become part of the problem.^11^

As nursing has evolved, so too has the nursing process—and it should continue to evolve. Modern nursing seeks to address the changing health care needs of our local and global societies, as well as of each person and population.^12^-^14^ In conjunction with nursing's evolution, we need to think critically about nursing's unique methodology—the nursing process. Perhaps the process can evolve to be utilized more in daily nursing practice; to better align with patient-centered goals of care; to be more capable of promoting patient collaboration through patient-friendly language; and to be better recognized by our medical colleagues and regulatory/payment organizations as a crucial contribution to health and well-being of patients, families, communities, and populations. (See *Who Is the Recipient of Nurses' Care*?^15^)

**Box 1:**
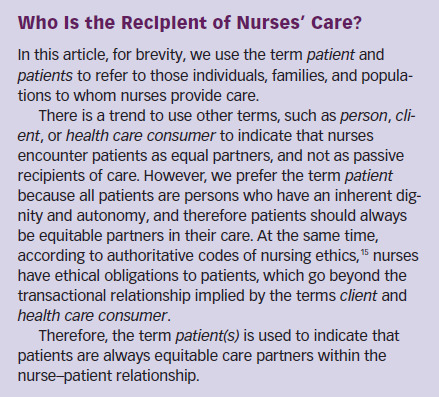
Who Is the Recipient of Nurses' Care?

This article proposes four suggestions for enhancing this process: (1) changing “nursing process” to “nursing's scientific method”; (2) adding two crucial steps—relationship building and communication—to the beginning and end of the method; (3) renaming the third and fourth steps from “nursing diagnosis” and “outcome identification” to “problems/strengths” and “goals”; and (4) emphasizing that nursing's scientific method is a critical thinking methodology. Evidence and research are cited to support these recommendations.

## CHANGE “NURSING PROCESS” TO “NURSING'S SCIENTIFIC METHOD”

Sometimes a term does not adequately connote the importance of a concept. This is true of the term *nursing process*. Although the nursing process is effective at guiding nursing practice, it is more than a process. We should call it what it is: nursing's scientific method. It is nursing's evidence-based scientific method that guides nurses on how to promote patient health and well-being.^3^

Nursing's scientific method reflects the scientific method,^16^ the goal of which is to guide critical thinking and decision-making and to mitigate errors and biases when seeking answers to questions. The scientific method (1) asks a question; (2) gathers data about what is known about the question; (3) forms a hypothesis about the answer to the question; (4) designs a methodology to test the hypothesis; (5) implements the methods to test and analyze the hypothesis; and (6) evaluates if the findings confirm the hypothesis, or if another hypothesis is needed to answer the question.^16^

Professional nursing practice is grounded in the scientific method. Nurses begin their care of patients by (1) asking the question, “What can I, as a nurse, do to promote this patient's health, comfort, and well-being?”; (2) assessing the patient's current status (gather information); (3) developing nursing diagnoses (hypotheses) about the patient's problems; (4) designing a plan (methodology) to promote and improve the patients' health/comfort/well-being; (5) implementing the plan (testing and analysis); and (6) evaluating the patient's outcomes (determine findings) to see if they achieved their desired health goals or if we need a reassessment. Using nursing's scientific method is crucial to professional nursing practice. When nurses do not employ it during their practice, they are not practicing professional nursing care.^11^

The term *nursing's scientific method* carries the connotation that nurses engage in the highly esteemed scientific method. This name change might inspire nurses to take this methodology more seriously and to use it more consistently. It might remind nurses to go beyond “following doctor's orders” and to fully engage in the unique contribution they can make to patients' health, comfort, and well-being through nursing's scientific method. Similarly, when interdisciplinary colleagues, reimbursement sources, and even patients hear that nurses employ nursing's scientific method, they may develop greater appreciation and esteem for nursing and the contribution nurses make to patient care.

## TWO NEW ELEMENTS

Evidence suggests that there are two additional elements that would enhance the patient-centeredness, effectiveness, and patient safety of nursing's traditional scientific method. One of these elements—relationship building—should begin before starting the assessment. The second—communication—should be the last element in nursing's scientific method as a reminder that nursing care is not complete until we communicate necessary patient information to patients, caregivers, and interdisciplinary team members. Both of these steps are highlighted as crucial to nursing practice in the core competencies for nursing education.^17^

**Relationship building**. Assessments, and each of the other steps of nursing's scientific method, are more effective when they take place within a warm, caring, and trusting nurse–patient/family relationship.^18^-^20^ Seeing each patient as a unique and valued person whose problems, concerns, and goals are of primary importance to the nurse is a core element of patient-centered care.^21^ The nature of the nurse–patient relationship has a direct impact on the quality of care the patient receives and the healing the patient achieves.^19^,^22^,^23^

In a first encounter, the nurse should approach the patient and the patient's family with courtesy, caring, and kindness.^20^ We should mindfully bring empathy and cultural sensitivity to the relationship and ensure good communication from the start; this includes meeting the patient's and family's unique language and health literacy needs.^24^ To ensure equitable care, we should consider and mitigate any biases or negative impressions we have about any of the patient's diversity groups.^25^ We can use words and behaviors to demonstrate we truly care about the patient.^23^ We should get to know the patient as a person by asking them what they care about, what their goals and concerns are, and what is most important to them.^26^ With this demonstration of caring as an introduction to care, patients tend to disclose important assessment information that they might otherwise conceal, even if the information is crucially important to the success of the care plan.^20^ Warm, caring, trusting relationships also set the stage for a collaborative care planning process. When nurses accept patients as equal trusted partners in care planning, patients tend to be more adherent and committed to following the care plan.^19^

**Communication**. Adding a communication element, which includes empathic communication with patients and families, interprofessional communication and collaboration, and documentation that supports interprofessional communication, contributes to the effectiveness and safety of patient care. Thus, communication is a crucial element in nursing's scientific method. Empathic communication with patients, families, and caregivers is characterized by kind and informative sharing, which promotes patient satisfaction with care.^27^,^28^ Good intraprofessional and interprofessional communication promotes patient safety and care effectiveness by reducing medical errors, substandard care, and missed care.^29^ Effective interprofessional collaboration results in better patient outcomes.^30^

Documentation should not only communicate information to the care team but also implicitly demonstrate how the nurse applied nursing's scientific method during the patient encounter. When it is obvious that the nurse used nursing's scientific method, nurses protect themselves and the organizations they work for against claims of malpractice.

## CHANGE “NURSING DIAGNOSIS” AND “OUTCOMES IDENTIFICATION” TO “PROBLEMS/STRENGTHS IDENTIFICATION” AND “GOALS”

One of the key elements of patient-centered care is that the nurse plans care in collaboration with the patient.^26^,^31^-^33^ Thus, nurses should partner with patients, and include their perspectives, in every element of nursing's scientific method. Understanding patients' perspectives about their health problems (nursing diagnosis) and what they want nurses to help them achieve (expected outcomes) is critically important to care plan effectiveness and patient satisfaction.^34^

A patient-centered way to include the patient in the care planning process would be to walk through nursing's scientific method with the patient. However, patients, and even nurses, may find the terms “nursing diagnosis” and “expected outcomes” a bit esoteric. These terms are nurse-centered terms, which conflict with our mission to promote patient-centered care planning.

Additionally, nurses are advised to avoid technical/scientific terminology when educating and working with patients. Using patient-friendly language, such as “problems/strengths identification” and “goals” instead of “nursing diagnosis” and “expected outcomes” may promote patient engagement and understanding of why and how the care plans they develop with nurses can help them. In turn, this engagement and understanding can promote patient adherence and better patient outcomes.^35^

Changing the name from diagnosis to problems/strengths does not mean that nurses should abandon the NANDA International (NANDA-I) diagnoses (previously North American Nursing Diagnosis Association list of diagnoses).^36^ These nursing diagnoses address the patients' experiences and responses to illness rather than focusing on the medical diagnoses.^10^ With their focus on symptom management and promoting well-being, most NANDA-I diagnoses will be readily understandable to patients.

## NURSING'S SCIENTIFIC METHOD AS A CRITICAL THINKING METHODOLOGY

The nursing process is a critical thinking methodology.^7^ According to the ANA, the nursing process is the patient-centered critical thinking model that guides nurses to use science-based and caring science methods for promoting patient health and well-being.^3^ Nurses who lack critical thinking skills are probably not using nursing's scientific method as they should.^11^

To think critically, people need an inquisitive, analytical attitude and a systematic structure.^11^ For nurses, this systematic structure is nursing's scientific method. It guides nurses to think purposely, critically, creatively, and effectively about how to guide patients toward their optimal health and well-being.^3^

Nurses analyze, evaluate, and interpret information at each stage of nursing's scientific method to inform our clinical judgments and decision-making.^10^ Nurses who use nursing's scientific process in this way are inquisitive, question their assumptions, explore different perspectives, consider root causes, and apply nursing and caring scientific evidence during each of the eight elements of nursing's methodology.

One difference between the traditional scientific method and nursing's scientific method is that in nursing, the method is less linear and more iterative and dynamic. This is because patients' needs, illnesses, and lives are dynamic, constantly changing and evolving. Although nursing's scientific method is conceptually modeled as a linear series of steps, we are constantly revisiting and jumping ahead to previous and future steps as we make new observations and learn new information through our encounters with the patient.^3^

For instance, during the assessment phase, nurses, using critical thinking, may discover an emergent situation requiring them to jump to the communication phase for urgent resolution of a medical problem. Or, during the implementation phase, nurses may detect a new problem that requires them to revisit the problem, goals, and planning phases. And frequently, during the evaluation phase, nurses discover that the goals are not being met with the current plan, which requires them to revisit all the different phases again. Critical thinking means nurses actively and reflectively think about their thinking processes during each patient encounter and each step of nursing's scientific process.

## NURSING'S EIGHT-STEP SCIENTIFIC METHOD

**Figure 1.**
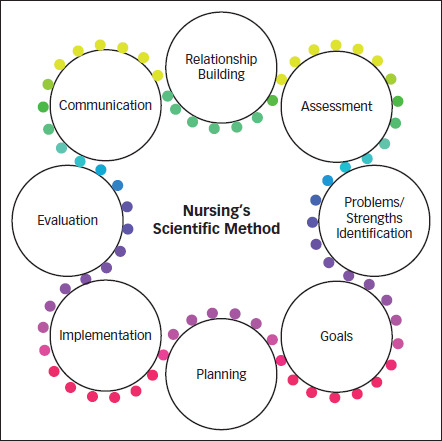
Nursing's Scientific Method

If we incorporate the recommendations above, the nursing process would evolve into something like the eight steps described below and shown in Figure 1.

**Relationship building**. One of the goals of this step is to form a warm, caring, and trusting relationship (the therapeutic relationship) with the patient. The other goal is to learn who the patient is as an individual person with unique concerns, priorities, and preferences. Both goals are fundamental to patient-centered care. If we do not accomplish these two goals during the initial stage of nursing's scientific method, we are not practicing patient-centered care. Critical reflective thinking will help the nurse to thoughtfully consider what this patient needs to feel comfortable and well cared for within the healing relationship the nurse seeks to establish. Although this is the first step in nursing's scientific method, it is iteratively incorporated into each of the other seven steps and into each patient encounter.

During this stage of nursing's scientific method, we should reflect on the following: How can I demonstrate that I value and care about this patient as a person? How can I show that I am trustworthy and understanding? How can I ensure that I am communicating and listening well to this patient and family? What questions should I ask to get to know this patient as a person?

**Assessment**. During assessment, the nurse investigates the patient's current status related to the patient's physical and mental–emotional health and well-being. The goal is to investigate factors that will affect the patient's outcomes, which may be amendable to nursing intervention. Depending on the setting, situation, and patient's individuality, the assessment will frequently need to expand beyond the traditional nursing assessment that typically focuses on physical, medical, and mental health problems.

Other factors that may need to be assessed include social determinants of health, culture, lifestyle, religion/spirituality, finances, learning needs, medication knowledge/management, motivation/confidence/ability to adhere to the care plan, family/caregiver accessibility/ability, and safety in facility and home environments, among other factors specific to each patient. For each patient, these factors will be different, and critical thinking skills are needed to determine assessment questions that dig deeper into factors that affect each unique patient's outcomes. During the assessment, we should ask: What factors will have an impact on this patient's ability to recover, maintain health, or achieve well-being? How can I best assess those factors?

**Problems/strengths identification**. During this phase, the nurse reflects carefully on the assessment data. The data are critically analyzed, summarized, and prioritized for issues that compromise the patient's health and well-being. The nurse strengthens the patient-centeredness of this step by also asking patients to share their perceptions of their problems, needs, and strengths. Inviting the patient to participate in this way can strengthen the patient's trust, self-efficacy, and adherence to the care plan. Identifying the patient's strengths with the patient—such as a supportive family, resilience despite adversity, or spiritual/religious beliefs—can also encourage patients' self-efficacy and adherence.^37^

The problem list should identify not only the physical/medical needs but also the nonmedical factors that compromise the patient's health and well-being. Thus, the problem list may include psychological, social, spiritual, financial, and knowledge-deficit problems among others as needed by individual patients. The official NANDA-I diagnoses can be used to identify patients' problems, or the nurse can list the symptoms, risk factors, and issues related to their medical diagnoses.

**Goals**. Although the nurse may have specific goals for the patient, if they are not congruent with the patient's health care goals, the care will not be patient centered, the patient is less likely to be adherent, and best outcomes are less likely.^38^ Nurses should understand what is important to the patient, and one way to do this is to understand what motivates the patient and what their health goals are. We can ask, “What are your health/health care goals and hopes?” When patients are not able to answer this question, another way to identify the patient's individualized goals is to ask, “What do your current problems keep you from doing that you would like to be doing?”

Nurses can collaborate with the patient to turn their hopes into goals that are SMART (Specific, patient can visualize the desired outcome; Measurable, nurse and patient can identify progress toward the goal; Achievable, the goal is feasible for this patient; Relevant, the goal is relevant to the patient's problems and needs; and Time-defined, how long the nurse/patient believe it will take to achieve the goal).^39^

**Planning**. Having identified the patient's current status (problems/strengths) and what the patient wants to achieve (goals), the nurse has a skeleton map for the nursing care plan. We know the starting point and the end we are trying to reach. We now ask: What are the best strategies for getting to where the patient wants to go? And what are the best strategies that will promote the patient's sense of well-being while working through the plan? Planning means we use our creativity and problem-solving skills to identify specific strategies (individualized to the patient's unique needs, preferences, culture, and lifestyle) that will enable the patient to meet their goals. Strategies are translated into the specific skills the nurse will use to achieve the goals.

The most effective care plans are the ones developed in collaboration with the patient. We can ask patients what strategies they think will help them reach their health goals. Sometimes patients have the most effective and innovative recommendations. We should incorporate their suggestions into the care plan. When suggesting strategies from our nursing knowledge, we can ask, “Do you think this will work? Can you think of some way to make this suggestion more helpful for you?” In order to create a patient-centered plan, we need to think critically, creatively, and flexibly with the patient about the best interventions for this patient.

**Implementation**. Once we have identified strategies and tasks that will help us meet the patient's goals for optimal health and well-being, we have “planned the work.” Now, in partnership with the patient, we “work the plan.” During this stage, we iteratively continue to strengthen the nurse–patient relationship, assess the patient, and consider if there are new problems and goals to consider.

**Evaluation**. Nurses should put their critical thinking skills into high gear when evaluating the effectiveness of the nursing care plan. We need to be open to changing the care plan when it seems it is not effectively meeting the patient's goals. As with all steps of nursing's scientific method, this step is taken in collaboration with the patient. We can ask the patient, “What do you think about the progress you are making in reaching your health care goals? Do you feel encouraged or discouraged about the progress you are making?”

Nurses should ask themselves: Is the plan effectively moving the patient toward their goals? Could the plan be more effective? Am I missing anything that is crucial to the patient's health and well-being? Do I need to dig deeper through a reassessment? Are the identified goals realistic for this patient?

**Communication**. At the end of a patient encounter, the nurse should think critically about who should be informed about new patient information; with whom the nurse should collaborate; and what needs to be documented to meet their patients' need for safe, high-quality, patient-centered care. Using critical reflection, we should ask ourselves: With whom should I *communicate* about what I just assessed and learned about the patient? With whom should I *collaborate* to promote safe and effective care? What needs to be *documented* so other team members have the information they need to ensure safe and effective care?

Concerned stakeholders interested in the patient's status and outcomes include the patient and the patient's family and caregivers. They also include physicians, nurse colleagues, rehab therapists, medical social workers, discharge planners, pharmacists, chaplains, insurance companies, and others important to the patient's health and well-being. When communicating with patients and family members, we need to ensure effective communication by attending to their unique communication needs related to language, literacy, and health literacy.^40^ The nurse should also consider the best way to communicate with each of the stakeholders: text messages, telephone calls, email messages, or electronic health record (EHR) documentation. Although communication, collaboration, and documentation should occur as needed through every step of nursing's scientific method, nurses should ensure that they have adequately communicated before thinking a patient encounter has been completed.

## CHALLENGES TO USING NURSING'S SCIENTIFIC METHOD

As every nurse knows, it can be difficult to incorporate nursing's scientific method into daily practice. The literature identifies several barriers. First, nurses do not always incorporate nursing's scientific method into their professional identity.^41^ All patient encounters should be grounded in nursing's way of thinking, problem-solving, and decision-making. Second, nurses frequently feel they do not have the time to do the deep and critical thinking needed to support patient-centered care by using nursing's scientific method.^42^ However, nurses can practice “thinking like a nurse” until the method is their automatic way of thinking. Third, EHRs frequently do not support nursing's scientific ways of thinking. They are often based on the medical model, making it difficult for nurses to document using nursing's scientific method.^43^ An important role for nursing leaders is to advocate for better ways to capture nursing's scientific method in EHRs.^44^

Despite challenges, we can commit to using nursing's scientific method. We can practice using it until it becomes the automatic, intuitive, and most efficient method for providing patient care on a daily basis. Using nursing's scientific method should become an intrinsic element of each nurse's professional identity.

## CONCLUSION

Nursing's scientific method supports nurses in their critical/creative thinking, problem-solving, and decision-making about how to provide patient-centered care. It is foundational to what makes a nurse a *professional* nurse. Developing skills in using nursing's scientific method for critical thinking begins in nursing school and gradually grows and develops through practice into an automatic, intuitive, and efficient way of thinking. Working collaboratively with the patient through the method can promote care that is truly patient centered and highly effective. Drawing on nursing knowledge and evidence-based practices through nursing's scientific method may help enhance the value medical colleagues and regulatory/payment organizations place on nursing's unique contribution to patient and population health and well-being.

The four recommended changes to the process nurses use to guide patient care are consistent with nursing's evolution into a more patient-centered and evidence-based way of thinking about and providing care.

## References

[1] Orlando IJ. *The Dynamic Nurse-Patient Relationship: Function, Process, and Principles*. Putnam; 1961. 2100331

[2] American Nurses Association. *Standards of Nursing Practice*. American Nurses Association; 1973.

[3] American Nurses Association. *Nursing: Scope and Standards of Practice*. 4th ed. American Nurses Association; 2021.

[4] Adraro Z Mengistu D. Implementation and factors affecting the nursing process among nurses working in selected government hospitals in Southwest Ethiopia. *BMC Nurs*. 2020;19(1):105. doi:10.1186/s12912-020-00498-8 33292177 10.1186/s12912-020-00498-8 PMC7654185

[5] Ito M Murakami K Ono S McMillan M. Reflections on critical thinking in the nursing process and Japanese nurse education. *J Probl Based Learn*. 2021;8(1):41–50. doi:10.24313/jpbl.2020.00318

[6] Lotfi M Zamanzadeh V Valizadeh L Khajehgoodari M Ebrahimpour Rezaei M Khalilzad MA. The implementation of the nursing process in lower-income countries: an integrative review. *Nurs Open*. 2020;7(1):42–57. doi:10.1002/nop2.410 31871690 10.1002/nop2.410 PMC6917928

[7] Mahmoud MH Bayoumy HM. Barriers and facilitators for execution of nursing process from nurses' perspective. *Int J Adv Res*. 2014;2(2):300–315.

[8] Zamanzadeh V Valizadeh L Tabrizi FJ Behshid M Lotfi M. Challenges associated with the implementation of the nursing process: a systematic review. *Iran J Nurs Midwifery Res*. 2015;20(4):411–419. doi:10.4103/1735-9066.161002 26257793 10.4103/1735-9066.161002 PMC4525336

[9] Shewangizaw Z. Determinants towards implementation of nursing process. *Am J Nurs Sci*. 2015;4(3):45. doi:10.11648/j.ajns.20150403.11

[10] Perez Rivas FJ Martin-Iglesias S Pacheco del Cerro JL Minguet Arenas C Garcia Lopez M Beamud Lagos M. Effectiveness of nursing process use in primary care. *Int J Nurs Knowl*. 2016;27(1):43–48. doi:10.1111/2047-3095.12073 25622991 10.1111/2047-3095.12073

[11] Falco-Pegueroles A Rodriguez-Martin D Ramos-Pozon S Zuriguel-Perez E. Critical thinking in nursing clinical practice, education and research: from attitudes to virtue. *Nurs Philos*. 2021;22(1):e12332. doi:10.1111/nup.12332 33029860 10.1111/nup.12332

[12] Backes DS Halmenschlager RR Cassola TP Erdmann AL Hamel K Costenaro RGS. Inseparability between public health, planetary health and the nursing process: premise for sustainable development. *Rev Esc Enferm USP*. 2024;58:e20240026. doi:10.1590/1980-220X-REEUSP-2024-0026en 38949513 10.1590/1980-220X-REEUSP-2024-0026en PMC11216715

[13] Institute of Medicine. *The Future of Nursing: Leading Change, Advancing Health*. 2011. Washington, DC: The National Academies Press. 24983041

[14] Toney-Butler TJ Thayer JM. *Nursing Process*. StatPearls Publishing; 2024. 29763112

[15] American Nurses Association. *Code of Ethics for Nurses with Interpretive Statements*. American Nurses Association; 2015.

[16] Grant H. *Scientific Method in Practice*. Cambridge University Press; 2003.

[17] American Association of Colleges of Nursing. *The Essentials: Core Competencies for Professional Nursing Education*. American Association of Colleges of Nursing; 2021.

[18] Feo R Conroy T Jangland E Towards a standardised definition for fundamental care: a modified Delphi study. *J Clin Nurs*. 2018;27(11-12):2285–2299. doi:10.1111/jocn.14247 29278437 10.1111/jocn.14247

[19] Molina-Mula J Gallo-Estrada J. Impact of nurse-patient relationship on quality of care and patient autonomy in decision-making. *Int J Environ Res Public Health*. 2020;17(3):835. doi:10.3390/ijerph17030835 32013108 10.3390/ijerph17030835 PMC7036952

[20] Narayan MC Mallinson RK. Transcultural nurse views on culture-sensitive/patient-centered assessment and care planning: a descriptive study. *J Transcult Nurs*. 2022;33(2):150–160. doi:10.1177/10436596211046986 34612735 10.1177/10436596211046986

[21] Tomaselli G Buttigieg SC Rosano A Cassar M Grima G. Person-centered care from a relational ethics perspective for the delivery of high quality and safe healthcare: a scoping review. *Front Public Health*. 2020;8:44. doi:10.3389/fpubh.2020.00044 32211362 10.3389/fpubh.2020.00044 PMC7067745

[22] Bahari Z Vosoghi N Ramazanzadeh N Moshfeghi S Aghamohammadi M. Patient trust in nurses: exploring the relationship with care quality and communication skills in emergency departments. *BMC Nurs*. 2024;23(1):595. doi:10.1186/s12912-024-02241-z 39183274 10.1186/s12912-024-02241-z PMC11345954

[23] Watson J. Caring science and human caring theory: transforming personal and professional practices of nursing and health care. *J Health Hum Serv Adm*. 2009;31(4):466–482. 19385422

[24] Howick J Kendrick A Anderson E. Does empathic care help and can it be taught. *Nurs Older People*. 2023;35(6):15–16. doi:10.7748/nop.35.6.15.s6

[25] Narayan MC. CE: Addressing implicit bias in nursing: a review. *Am J Nurs*. 2019;119(7):36–43. doi:10.1097/01.NAJ.0000569340.27659.5a 10.1097/01.NAJ.0000569340.27659.5a 31180913

[26] Narayan MC. What constitutes patient-centered care in home care? A descriptive study of home health nurses' attitudes, knowledge, and skills. *Home Healthc Now*. 2022;40(6):317–329. doi:10.1097/NHH.0000000000001124

[27] Kalaja R. Determinants of patient satisfaction with health care: a literature review. *Eur J Nat Sci Med*. 2023;6(1):43–54. doi:10.2478/ejnsm-2023–0005

[28] Priyantini D. The correlation of therapeutic communication in nursing and patient satisfaction during caring in hospital. *Indones Nurs J Educ Clin*. 2023;8(1):67–75.

[29] Institute of Medicine. *To Err Is Human: Building a Safer Health System*. National Academies Press; 2000. 25077248

[30] Wei H Horns P Sears SF Huang K Smith CM Wei TL. A systematic meta-review of systematic reviews about interprofessional collaboration: facilitators, barriers, and outcomes. *J Interprof Care*. 2022;36(5):735–749. doi:10.1080/13561820.2021.1973975 35129041 10.1080/13561820.2021.1973975

[31] Fix GM VanDeusen Lukas C Bolton RE. Patient-centred care is a way of doing things: how healthcare employees conceptualize patient-centred care. *Health Expect*. 2018;21(1):300–307. doi:10.1111/hex.12615 28841264 10.1111/hex.12615 PMC5750758

[32] Frampton S Guastllo S Brady C *Patient-Centered Care Improvement Guide*. Planetree Inc. and Picker Institute. 2008.

[33] Registered Nurses' Association of Ontario. *Person- and Family-Centered Care: Clinical Best Practice Guideline*. 2015. https://rnao.ca/sites/rnao-ca/files/FINAL_Web_Version_0.pdf

[34] Mielke K Frerichs W Collen K Lindig A Harter M Scholl I. Perspective on patient-centered communication: a focus group study investigating the experiences and needs of nursing professionals. *BMC Nurs*. 2024;23(1):822. doi:10.1186/s12912-024-02487-7 39533302 10.1186/s12912-024-02487-7 PMC11558982

[35] Graffigna G Barello S Bonanomi A. The role of patient health engagement model (PHE-model) in affecting patient activation and medication adherence: a structural equation model. *PLoS One*. 2017;12(6):e0179865. doi:10.1371/journal.pone.0179865. 28654686 10.1371/journal.pone.0179865 PMC5487073

[36] NANDA International. *Nursing Diagnoses: Definitions and Classification*. 2024.

[37] Al Hashmi I Al Omari O. Self-efficacy in relation to adherence to healthy behaviours among pregnant women: a concept analysis. *Cent Eur J Nurs Midwifery*. 2022;13(2). doi:10.15452/cejnm.2021.12.0003

[38] Kwame A Petrucka PM. A literature-based study of patient-centered care and communication in nurse-patient interactions: barriers, facilitators, and the way forward. *BMC Nurs*. 2021;20(1):158. doi:10.1186/s12912-021-00684-2 34479560 10.1186/s12912-021-00684-2 PMC8414690

[39] Ogbeiwi O. General concepts of goals and goal-setting in healthcare: a narrative review. *J Manage Organ*. 2018;27(2):1–18. doi:10.1017/jmo.2018.11

[40] Do V Asim A Coffey M Mahant S. Improving health outcomes for patients and families with preferred language other than English or French (PLOEF). *Paediatr Child Health*. 2024;29(4):211–213. doi:10.1093/pch/pxad017 39045473 10.1093/pch/pxad017 PMC11261821

[41] Shahzeydi A Abazari P Gorji-Varnosfaderani F Ashouri E Abolhassani S Sabohi F. Breaking the taboo of using the nursing process: lived experiences of nursing students and faculty members. *BMC Nurs*. 2024;23(1):621. doi:10.1186/s12912-024-02233-z 39237916 10.1186/s12912-024-02233-z PMC11378630

[42] Narayan MC. Structural barriers to high-quality home healthcare nursing: what home health nurses want Medicare policy makers and agency administrators to know. *Home Health Care Manage Pract*. 2023;36(1). doi:10.1177/1084822323118059

[43] Hants L Bail K Paterson C. Clinical decision-making and the nursing process in digital health systems: an integrated systematic review. *J Clin Nurs*. 2023;32(19-20):7010–7035. doi:10.1111/jocn.16823 37485751 10.1111/jocn.16823

[44] Bar KA Lima BDS Martelle GM Silva SCD Santos MRD Costenaro RGS. Nurses' perception of the nursing process and its relationship with leadership. *Rev Bras Enferm*. 2024;77(1):e20230371. doi:10.1590/0034-7167-2023-0371 38655980 10.1590/0034-7167-2023-0371 PMC11034378

